# Automatic Identification of Analogue Series from Large Compound Data Sets: Methods and Applications

**DOI:** 10.3390/molecules26175291

**Published:** 2021-08-31

**Authors:** José J. Naveja, Martin Vogt

**Affiliations:** 1 Instituto de Química, Universidad Nacional Autónoma de México, Mexico City 04510, Mexico; navejaromero@gmail.com; 2 Department of Life Science Informatics, B-IT, LIMES Program Unit Chemical Biology and Medicinal Chemistry, Rheinische Friedrich Wilhelms-Universität, Friedrich-Hirzebruch-Allee 5-6, 53115 Bonn, Germany

**Keywords:** analogue series, compound-core relationships, core structure, matched molecular pairs, matched molecular series, molecular scaffold, structure-activity relationships, medicinal chemistry, cheminformatics

## Abstract

Analogue series play a key role in drug discovery. They arise naturally in lead optimization efforts where analogues are explored based on one or a few core structures. However, it is much harder to accurately identify and extract pairs or series of analogue molecules in large compound databases with no predefined core structures. This methodological review outlines the most common and recent methodological developments to automatically identify analogue series in large libraries. Initial approaches focused on using predefined rules to extract scaffold structures, such as the popular Bemis–Murcko scaffold. Later on, the matched molecular pair concept led to efficient algorithms to identify similar compounds sharing a common core structure by exploring many putative scaffolds for each compound. Further developments of these ideas yielded, on the one hand, approaches for hierarchical scaffold decomposition and, on the other hand, algorithms for the extraction of analogue series based on single-site modifications (so-called matched molecular series) by exploring potential scaffold structures based on systematic molecule fragmentation. Eventually, further development of these approaches resulted in methods for extracting analogue series defined by a single core structure with several substitution sites that allow convenient representations, such as R-group tables. These methods enable the efficient analysis of large data sets with hundreds of thousands or even millions of compounds and have spawned many related methodological developments.

## 1. Introduction

An analogue series is a set of compounds sharing a common core structure with different substitutions at one or more substitution sites. In many cases, it admits R-group table representations detailing the fragments at individual substitution sites. However, molecules showing only minor variations in the core structure might also be considered part of the same analogue series. Although analogue series arise naturally and are systematically explored during lead optimization efforts, identifying potential analogue compounds and analogue series in large, potentially very diverse compound databases is much more challenging and non-trivial [1,2,3].

Analogue series play a key role in drug discovery. They form the basis for lead optimization efforts to improve activity, ADMET properties, and other endpoints through minor structural changes [4]. As such, they are very rich in information on structure-activity relationships (SAR) and can offer insights into structural determinants relevant for biological properties, such as target-specific activity [5,6,7,8,9].

Modern databases, such as ChEMBL [10] or PubChem [11], contain millions of compounds with associated activity and property annotations. Therefore, systematically searching for analogue series in such large compound data sets promises to be a rich source for SAR information, reaching far beyond investigating individual analogue series for specific optimization campaigns.

Initial efforts have used scaffold decomposition methods—based, for example, on the Bemis–Murcko scaffold definition [12]—to identify analogue series, which later gave rise to hierarchical scaffold decomposition methods, such as the scaffold tree [13]. Over the last two decades, several new techniques and algorithms have been designed to comprehensively analyze large compound data sets for a data-driven identification of analogue series without relying on a fixed single scaffold decomposition for each molecule, but instead imposing some restrictions on analogue series by defining specific rules for a scaffold-based decomposition [1,2,3,14]. A central concept to many of these approaches is the systematic fragmentation of individual molecules to identify putative core structures and group molecules by common cores. Nonetheless, these methods often apply different definitions for identifying valid core structures [1,2,3,14].

This methodological review outlines the most popular and recent developments to automatically identify analogue series from large libraries. We first focus on fundamental concepts and basic algorithms, review scaffold-based approaches, and introduce matched molecular pairs (MMPs) and the fragment and index approach that is the basis of subsequent algorithmic developments. Afterward, we present additional methodologies, such as the SAR matrix and the compound-core relationship (CCR) approach; the latter allows identifying analogue series based on core structures with multiple substitution sites. Finally, an exemplary analysis of a collection of PPAR agonists highlights some of the methodologies presented.

## 2. Fundamental Concepts and Algorithms

### 2.1. Molecular Similarity

Within the computational study of structure-activity relationships, the concept of molecular similarity and its quantification play a central role [15]. In contrast to bioactivity, which can be experimentally measured—for instance, by estimating inhibition coefficients or binding affinities—the molecular similarity is, in essence, a concept dependent on the viewpoint and application. There are many approaches to molecular similarity that are based on either molecular shape of three-dimensional conformations, two-dimensional chemical structures, shared pharmacophoric patterns, or shared physicochemical or biological properties, as well as approaches based on numerical descriptor representations identifying molecular properties or structural elements using fingerprints [15,16,17].

For large-scale analysis, the quantification of molecular similarity using fingerprint- or descriptor-based methods is computationally efficient and accessible. However, it does not translate directly to structural similarity as perceived by the medicinal chemist. On the other hand, structural definitions of molecular similarity based on, for instance, the maximum common subgraph or on compounds sharing a common scaffold are hard to define in a rigorous, algorithmically accessible manner. Moreover, they are often either computationally costly, thus limiting their application to large compound data sets, or are constrained to a predefined set of core structures [12,18,19,20].

The major limiting factor of approaches relying on pairwise comparisons, such as fingerprint methods or maximum common subgraphs, is that these have to be applied to each possible pair of compounds in a database, thus resulting in quadratic runtime relative to the database size. This is already challenging for databases comprising tens of thousands of compounds, but it becomes infeasible for databases of hundreds of thousands or millions of compounds without smart pre-filtering techniques [21].

### 2.2. Bemis–Murcko Scaffolds and Cyclic Skeletons

Bemis and Murcko presented the first formal definition of scaffolds in 1996 [12]. Compounds are represented as ring systems, linker chains connecting ring systems, and acyclic terminal side chains that can be represented as R-groups. A *scaffold* is then defined as the combination of the ring systems and linker chains. This scaffold concept can be further generalized by considering graph frameworks [12], also termed cyclic skeletons [20], that only consider the topological graph structure and omit atom types and bond orders. A final abstraction disregards ring sizes and linker chain lengths, resulting in reduced cyclic skeletons. Xu and Johnson [20] used these scaffold definitions to group molecules into “molecular equivalence classes” in 2001. This represents an early adoption of the invariant principle to classify compounds into analogue series or, more generally, into series sharing some well-defined topological characteristics.

As such, scaffold R-group decompositions based on the Bemis–Murcko scaffold do not allow any ring substitutions. However, ring-containing substituents in analogue series exploration, for instance, during lead optimization efforts, are quite prevalent. Again, this is a manifestation of the observation that there is no unambiguous general way to define a molecule’s scaffold; it rather depends on the biological context under consideration or the synthetic accessibility [22]. This issue has been addressed in more recent developments that decompose a molecule not only into one but into many putative analogue-defining core structures, which allows organizing molecules into pairs or series with a common core. This more general approach allows a single molecule to be part of multiple series/pairs based on different scaffolds, thus encouraging the exploration of SARs from different viewpoints using, for instance, SAR matrix representations [23].

Fundamentally, most algorithms for identifying analogue series today can be traced back to the matched molecular pair (MMP) concept and the algorithm introduced by Hussain and Rea in 2010 [14], which will be discussed next.

### 2.3. Matched Molecular Pairs

**Figure 1:**
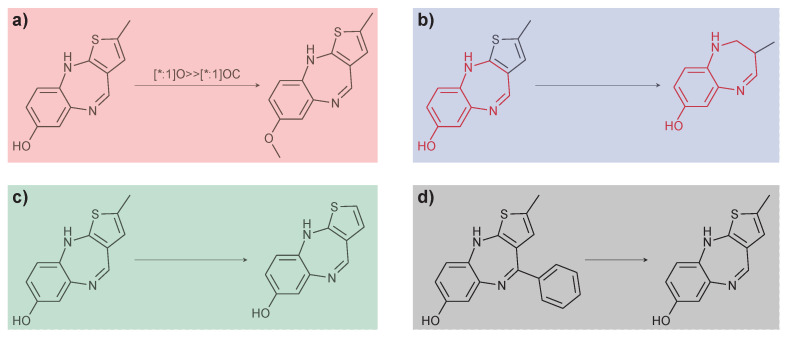
Exemplary MMPs with different definitions: (**a**) transformation-based; (**b**) maximum common subgraph; (**c**) exhaustive fragmentation, and (**d**) retrosynthetic fragmentation.

The MMP formalism is a structural definition of molecular similarity. The expression was coined by Kenny and Sadowski [24], who considered pairs of compounds related to each other by a single (predefined) transformation [25]. Many current approaches define an MMP as a pair of compounds related to each other by a small structural change at a single site (see Figure 1).The traceability provided by limited structural differences makes the SAR insights obtained through MMP analysis (MMPA) more intuitive than those produced by many other similarity-based analyses [26]. For example, MMPA can systematically capture the chemical knowledge in a database and quantify the average effect of a given transformation [27,28,29].

According to context and practical considerations for algorithmic identification, approaches for identifying MMPs can be roughly organized into three different categories. First, MMPs can be defined based on predefined chemical transformations that would convert one compound into the other (see Figure 1a) [21,24,25,30]. A variation of this approach uses a set of predefined substructures instead of chemical transformations and reduces the problem to a substructure search [25]. Second, MMPs can be determined through the maximum common substructure (MCS), restricting the difference of two molecules to a single substructure (see Figure 1b) [18,19]. While the first approach has low computational complexity, it is limited to predefined transformations or substructures. In contrast, the second approach is not limited in this way but requires pairwise computations; pre-filtering methodologies can limit the number of pairwise comparisons to reduce computational load. A third, conceptually different approach systematically applies fragmentation rules to each molecule: a pair is considered an MMP if it is possible to reduce both compounds to the same core structure (see Figure 1c,d) [14]. This approach is computationally efficient, especially for large data sets, without relying on predefined transformations. However, transformations of MMPs using this approach are typically limited to complete rings and ring systems; small hetero-atom ring substitutions are not detected directly. Table 1 summarizes these approaches. For an extensive review of MMP algorithms, see Reference [26] or Reference [28].

The three algorithmic MMP definitions above are arguably the most widely used. Nonetheless, other MMP definitions are available and helpful when working with specific problems. For example, matched peptide analysis optimizes MMPA for studying peptides by considering changes in the amino acid sequence rather than atom changes [31]. Other approaches, such as OOMMPPAA and Wonka, include three-dimensional information in MMPA for abstracting pharmacophoric knowledge from protein-ligand complexes [32,33]. Another related approach is fuzzy matched pairs, where molecules are encoded into pharmacophoric patterns [34]. Combining some of the methods above leads to other strategies aiming at more comprehensive results [19,35].

For the automated identification of analogue compounds in large databases, efficiency is a central concern. Notably, the fragmentation-based approach systematically explores all possibilities by which a compound splits into a core (scaffold) and fragment structure(s). Thus, the computational burden is shifted to individual compounds and will only increase linearly with increasing compound data set size.

### 2.4. Fragment-and-Index Approach: From Matched Molecular Pairs to Series and Scaffolds

The fragment-and-index approach as introduced in Reference [14] for the identification of MMPs refers to the process of first fragmenting molecules, possibly in many different ways, and using canonical representations of the core/scaffold structures obtained from these fragmentation steps as indices or keys to group molecules. The method requires rules to identify potential cuts in molecules and criteria for fragmented parts to be considered legitimate core structures and valid substituent fragments.

For efficiency reasons, hydrogen atoms are not regarded as valid fragments, and only bonds between non-hydrogen atoms are considered initially: potential MMPs involving hydrogen fragments are treated separately. One rule common to all fragmentation algorithms is that a cut, i.e., removing a bond, results in a separation of a molecule into two fragments, and fragmentation can occur at all acyclic single bonds or a chemically meaningful subset of bonds [14,26,37]. Thus, the single-site transformation defining an MMP is restricted to complete ring systems and does not include, for instance, simple hetero-atom substitutions within rings directly. One specific variation of this approach takes the synthetic accessibility of molecules into account. It will cut bonds only along retrosynthetic rules, such as those defined by the retrosynthetic combinatorial analysis procedure (RECAP) [38], in order to enhance synthetic interpretability [37,39], which results in a reduced set of so-called “cuttable” bonds. A comparison of both approaches for MMPs showed that RECAP MMPs represent about half of the total MMPs in a typical database [37].

**Figure 2:**
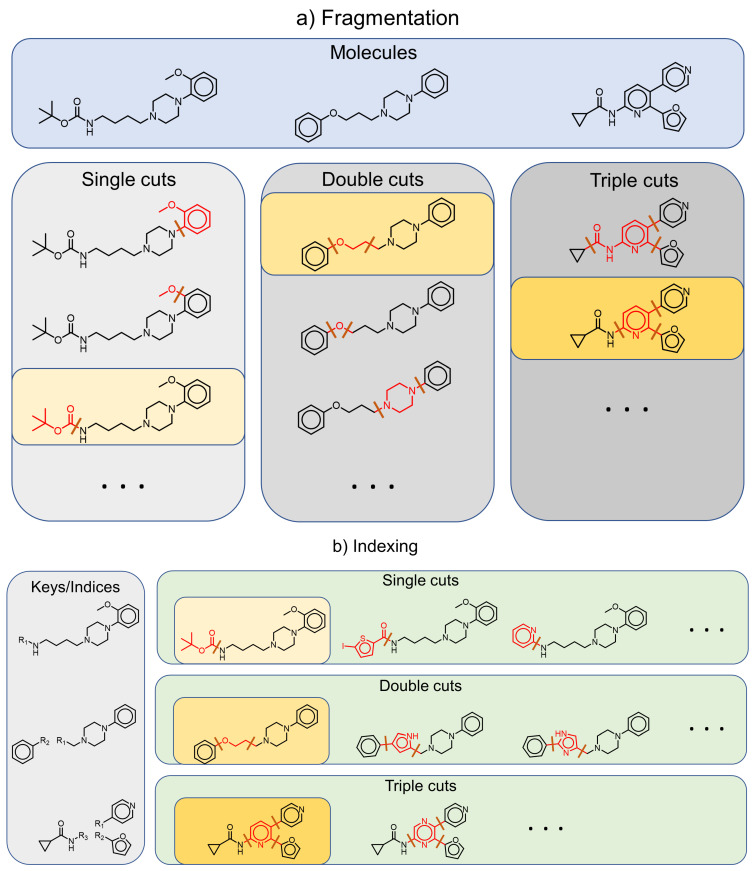
The fragmentation-and-index approach for matched molecular pairs/series. (**a**) The fragmentation step of the Hussain and Rea MMP algorithm will perform systematic single, double, and triple cuts for each molecule and identify small fragments (in red) that are connected by either one, two, or three bonds to the remainder of the molecule (in black), a potentially disconnected “core structure”. (**b**) During indexing, these core structures are used as keys or indices and all fragmentations are organized by their keys. For the fragmentations highlighted in yellow, an exemplary index table is shown containing fragmentations from other molecules with corresponding keys. All molecules associated with fragmentations sharing the same core structure form an MMS and each pair of this series forms an MMP.

In the original formulation by Hussain and Rea [14], the transformation fragments of an MMP are connected by one, two, or three bonds to the common core structure of the molecule (see Figure 2). The consideration of up to three simultaneous cuts in a single molecule can cause efficiency issues for large molecules. Note that double and triple cuts result in MMPs where the common core structure consists of two or three disconnected fragments. By restricting the approach to a single cut, only MMPs are identified that differ by a single terminal fragment corresponding to a decomposition of the molecule into a connected scaffold structure and a single R-group substitution [26]. Furthermore, concrete implementations might apply some restrictions on the admissible molecule size, as well as admissible fragment size (e.g., ten heavy atoms [14]), and requirements that the exchanged fragment is small compared to the overall molecule size [1,40]. Moreover, using sets of chemically meaningful transformations defined a priori reduces computation time and leads to more interpretable results. Specifically, RECAP rules result in chemically intuitive MMPs and have been extensively studied in SAR analysis [37,39].

In some cases, one of the molecules in an MMP has an “empty” substitution containing no heavy atoms at an R-group. Given that only cuts between non-hydrogen atoms are considered, MMPs involving such hydrogen fragments cannot be detected directly. Instead, hydrogen substitutions are identified in a post-processing step by searching for molecules in the database corresponding to a hydrogen-substituted core structure [14,41].

The core structures obtained in fragmentation steps correspond to unique “core indices”. Canonical SMILES are optimal representations for core indices [29]. Core indices can then be used to organize all fragmentations of all molecules of a data set. Thus, all molecules possessing a fragmentation with a common core will be clustered and form what is known as a matched or matching molecular series (MMS) [1,42]. Any pair of compounds from this series forms an MMP.

The exhaustive exploration of possible fragmentations can lead to ambiguous multiple common core structures shared in an MMP, all of which are identifiable through the fragment-and-index approach. Often, only the fragmentation with the largest core structure will be retained as representative of the MMP [40]. An extensive discussion of the fragment-and-index approach for MMP identification, including subtle issues, such as the algorithmic treatment of stereochemistry, can be found in Reference [29].

## 3. Methodological Developments Related to the MMP Concept and Scaffold Identification

Scaffold and R-group analysis remains a fundamental part of SAR exploration in medicinal chemistry. Therefore, extending the MMP formalism for organizing annotated chemical libraries into analogue series, identifying distinct scaffolds and R-groups substitutions, and identifying analogue series with R-group substitutions at multiple sites has been the focus of algorithmic extensions.

### 3.1. SAR Transfer and SAR Matrix

As described above, the fragment-and index approach will not only identify pairs of compounds but instead all compounds of a data set sharing a common scaffold structure and organize them into an MMS, i.e., an analogue series with a single scaffold and substitutions at a single site [1,42]. Such analogue series with limited variations provide a solid basis for the study and interpretation of SARs and led toward SAR transfer studies [43,44,44] and methodological extensions, such as the SAR matrix [23,45,46,47,48].

The MMS approach allows the identification of pairs of analogue series with overlapping substituents. SAR transfer refers to the notion that the same substituents in two series show similar potency progression against a given target and, thus, allows the inference of potency progression from one series to another. This concept can also be extended to the study of multi-target potency progression [49].

Methodologically, the SAR matrix represents an extension of this approach [23]: core structures of MMS that themselves form MMP relationships are organized in rows, and columns represent substitution fragments of the individual MMS (see Section 5 for an exemplary SAR matrix). The original publication modified the fragment and index approach to allow fragment substitutions at up to three different sites [23,50]. Therefore, it is a precursor to the more general CCR approach discussed below. SAR matrices are appropriate for the study of (single-site) substitutions in related core structures identifying structurally related compounds rich in SAR information and can serve as an analytical tool for exploring single and multi-target SARs. They are also helpful in potency prediction [45,51] using a Free-Wilson [52] approach and for prospective compound design [47]. In Reference [53], SAR matrices augmented with a molecular grid view that represents real and virtual compounds have been extended to an activity landscape representation aiding in the large-scale analysis of data sets beyond single SAR matrices, as well as in compound selection for prospective applications.

An alternative approach to SAR matrices was proposed by Agrafiotis et al. [5] using manual or MCS-based approaches to identify common core structures of a series. Based on an R-group analysis, a matrix-like representation termed SAR maps was introduced, where cells represent compounds and rows and columns represent substitutions at different sites [5]. A variation of this method termed single R-group polymorphisms (SRPs) uses a matrix approach, where rows and columns represent two different shifts at a single substitution site, and the cells record the average potency difference observed for a single analogue series [54]. SRPs study the SAR of single-site substitutions in the same scaffold while varying substitutions at other sites.

### 3.2. Networks and Analogue Series-Based Scaffolds

The original MMP approach, described in Section 2.4, set the foundations for the identification of more extensive structurally related series that are not necessarily defined by a single core structure and a single substitution site. To this end, MMPs and MMSs have been organized into networks in different ways. For example, Wawer and Bajorath [1] introduced the bipartite matched molecular series graph (BMMSG) for SAR analysis. A BMMSGs describes MMP fragmentations as graphs where molecule nodes are connected to index nodes representing the core structures.

Chemical space networks (CSN) [55,56] are network representations where nodes correspond to molecules and edges connect molecules satisfying a predetermined similarity criterion. In Reference [57], CSNs for compounds with a target-specific activity have been investigated based on MMP relationships. Conceptually, MMP-CSNs are network projections of the BMMSGs, where index nodes have been eliminated by directly adding edges between molecules connected to the same index node. The intuitive nature of MMP relationships makes MMP-CSNs attractive for SAR analysis; for instance, the concept of coordinated activity cliffs, i.e., sets of structurally similar molecules with significant pairwise potency differences forming tightly connected clusters, originated from MMP-CSN analysis [40].

MMP-CSNs tend to organize data sets into separate communities of molecules, where each community consists of structurally similar compounds defining an analogue series. This approach to analogue series identification has been explored in References [2,58]. Typically, these MMP-CSNs consider only single-cut MMPs, thus facilitating the interpretation of network clusters as structural analogue series obtained by R-group substitutions. For some of these series, a common core fragment can be identified, giving rise to the concept of the analogue series-based scaffold (ASBS) [2,58]. An ASBS emerges from subsets of molecules with more interpretable SARs [59] that are not limited to single-site substitutions.

### 3.3. Compound-Core Relationships

By definition, ASBSs are only valid for analogue series where a common substructure is representative of the scaffold of every molecule. However, connected components of MMP networks can contain divergent scaffolds for large data sets induced by a dense local coverage of chemical space, i.e., data sets with continuous variations of scaffolds representing advanced exploration of analogues [36]. While the ASBS approach can identify scaffolds with multiple substitution sites, it does not systematically explore all such potential scaffold structures. The recently developed compound-core relationship (CCR) approach addresses this issue through modification of the MMP fragmentation procedure [3].

**Figure 3:**
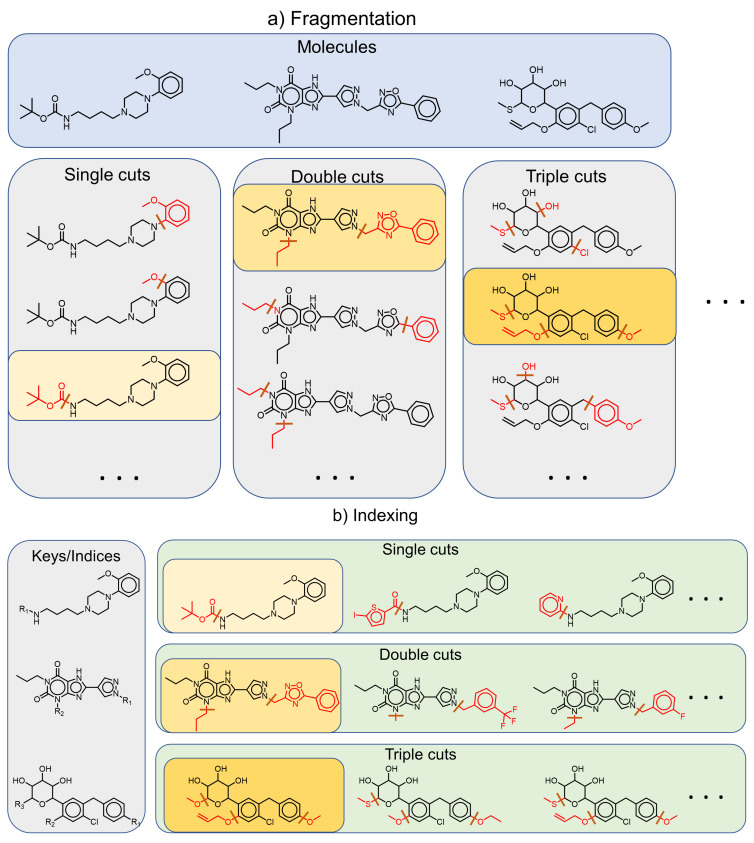
The fragmentation-and-index approach for the compound core relationship approach. (**a**) During fragmentation, all molecules are systematically cut at one, two, three, or more bonds (up to a predefined limit), thus splitting a molecule into a single connected core structure and one or more terminal substituents. (**b**) During indexing, fragmentations are organized by their core structure. For the fragmentations highlighted in yellow, an exemplary index table is shown below. Compounds that share a core structure are grouped together and form an analogue series with one, two, three, or more substituents. For single cuts the approach is identical to the MMP method (see Figure 2). However, for multiple cuts, the CCR approach results in scaffolds with multiple terminal substituents.

**Figure 4:**
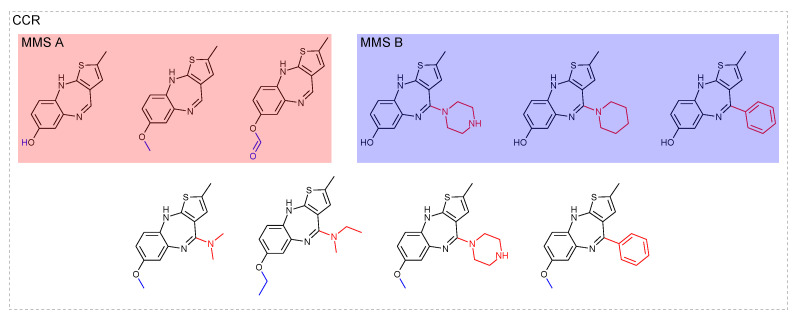
Comparing CCR versus MMS. MMS are usually defined on the basis of the single cut MMP. This makes it difficult to cluster molecules with multiple substitution sites. The CCR formalism allows defining cores through multiple cuts, as long as the core is a connected substructure of significant size (for example, at least two-thirds of the total molecule).

**Figure 5:**
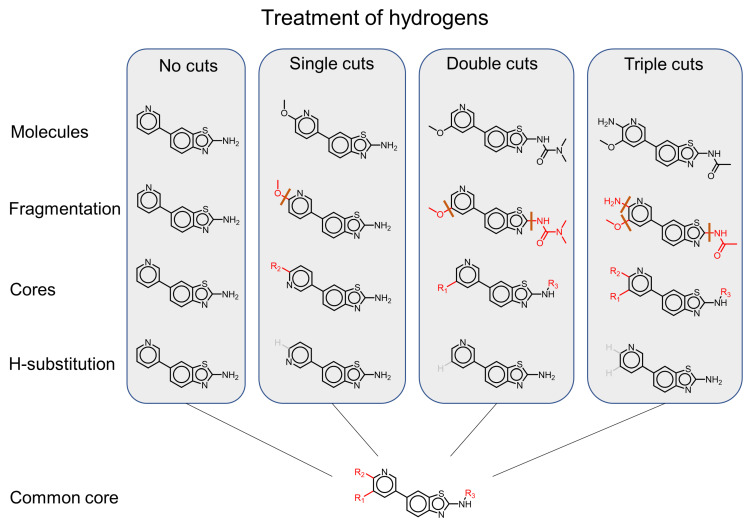
Treatment of hydrogen cuts. For each fragmentation of a molecule, a hydrogen-substituted core structure is obtained by replacing each attachment point with a hydrogen. If no cuts are performed for a molecule, no hydrogen substitution is necessary, and the molecule itself is its hydrogen-substituted core. All fragmentations with a common hydrogen substituted core are ultimately grouped together and form a single analog series with multiple substitution sites.

The CCR approach aims at identifying structural analogues characterized by a single core structure with multiple substitution sites. To this end, systematic fragmentation of compounds at one or multiple sites produces a single connected core structure with one or more R-groups substituents (see Figure 3). This deviation from the original fragmentation approach of Hussain and Rea [14] enables the method to detect variations of a molecule at multiple sites; however, these sites are restricted to terminal fragments, in contrast to the original formulation that can detect a single variation at a non-terminal site (see Figure 4). In its original formulation, the CCR approach was implemented using retrosynthetic fragmentation rules, a restriction aimed at generating chemically feasible analogue series with multiple substitution sites. In addition, the CCR method introduced the concept of a “hydrogen-substituted core structure” where hydrogens replace all substitutions sites of the core scaffold of a fragmentation. By grouping all fragmentations with a common hydrogen-substituted core structure together, analogue series with substitutions at different sites emerge (see Figure 5). This process can result in scaffolds with a nominally large number of substitution sites but a limited number of non-hydrogen substitutions. Ultimately, such series can be represented by R-group tables [3].

The extensive fragmentation of the CCR methods can result in many overlapping analogue series where shared compounds have been fragmented into core scaffolds of different sizes and with a different number of cuts. Such scaffolds can be organized into a network representation where edges between scaffolds represent compounds or compound sets that can be fragmented into both scaffolds [3,9]. Further processing of the overlapping sets of analogue series can also be applied to partition a data set into disjoint sets of analogue series by preferably assigning compounds that are part of multiple series to the larger ones [3].

### 3.4. Scaffold-Based Approaches

In order to assess the structural diversity of molecule data sets, scaffold structures can be investigated systematically. For specific analogue series, MCS-based approaches are feasible for performing R-group decompositions. For example, in Reference [60], the directed R-group combination graph represents R-groups tables in a directed graph structure, based on the substituents for each analogue. The AnalogExplorer revisited this strategy: analogue series were classified based on the Bemis–Murcko scaffold followed by MCS identification and R-group decomposition, and subsequently visualized as R-group trees where branches represent particular substitutions [6]. A second version of the AnalogExplorer took stereochemical information into account [61].

The MMP algorithm and the extensions discussed above readily provide an initial method for scaffold identification. Alternatively, rule-based approaches, such as the one pioneered by Bemis–Murcko [12] and Xu and Johnson [20], can be used for the hierarchical classification of molecules and scaffolds into different topological chemotypes [62]. This basic strategy has been refined in several approaches by iterative fragmentation of rings that create a hierarchy of scaffolds organized in a tree-like structure, where each scaffold is assigned a single parent scaffold that is a substructure of the child [13,63]. Based on this principle, recent interactive hierarchical scaffold explorers allow the definition of a desired scaffold hierarchy [64,65]. Subsequently, scaffolds at different levels of the hierarchy can be used as the basis for the extraction of analogue series. Furthermore, the parent definition can be relaxed to allow multiple parent scaffolds, resulting in more complex networks [66,67]. The analysis of such networks can be facilitated by enrichment analysis of frequently occurring scaffolds and by pruning away infrequent scaffolds. The RDKit-based *rdScaffoldNetwork* implementation is a flexible tool for the generation of scaffold networks with the option of applying different fragmentation rule sets [68].

An essentially non-hierarchical network approach was introduced in Reference [36]. Here, the CCR methodology was used to identify scaffolds of analogue series. Each scaffold node in a network represents one analogue series of compounds sharing this substructure. An edge connects scaffold nodes in a network if their analogue series share one or more compounds. Notably, scaffolds in a network cluster vary in size and can represent smaller analogue sets where one or more moieties are fixed. This concept has been extended in so-called constellation plots, where core structures are mapped onto a two-dimensional chemical space representation using descriptor representations and low-dimensional projection methods. The constellations are then formed by edges connecting cores if a molecule in the data set matches both of them [9]. For an example, see Section 5.

## 4. Exemplary Applications

### 4.1. Analogue Screening and Virtual Analogues

Madariaga-Mazón et al. [69] curated a database of 336 molecules isolated from plants used in traditional medicine against *diabetes mellitus* type 2. Considering that natural products and their analogues can be hard to synthesize, the database was extended using a virtual screening methodology using the CCR method to search for analogues of any of the molecules in ZINC 15 [70], an ultra-large chemical library of commercially available compounds. This procedure led to a 23-fold expansion of the database solely with compounds that are potentially purchasable [69].

The fragmentation process described in Section 2.4 systematically generates core and fragment structures that can also be used for the generation of virtual analogues and has been applied in several studies. The “chemical reasonable mutations” (CReM) approach [71] considers the local context of MMP-based fragmentations to enhance synthetic accessibility. Virtual chemical space is then explored by random “mutations” replacing fragment replacements, molecular growth, i.e., replacing hydrogens with larger fragments, and by linking fragments. By design, the method produces valid chemical structures and can also control the synthetic complexity of the generated molecules. Since the applied fragmentation steps will not split complete ring systems, one drawback of the method compared to deep neural network approaches, such as variational auto-encoders [72], is that no compounds with novel ring systems can be generated.

Yoshimori and Bajorath [51] utilized SAR matrices for the identification of analogue and virtual analogue compounds that can be constructed by recombination of core and substituent fragments of SAR matrices. This idea was further developed in the DeepSARM methodology that combines SAR matrices with generative deep neural networks to generate focused libraries for a single or dual specific biological targets [51,73,74].

The exploration of the local chemical space around a given analogue series through systematic fragmentation was presented in Reference [75] based on MMP transformations. In References [76,77], virtual compounds are generated by fragment recombination using RECAP-MMPs to assess the chemical space coverage of analogue series. Furthermore, the papers introduced a methodology to quantify the saturation level of analogue series and assess their potential for further SAR progression (i.e., the identification of compounds with improved endpoints) in lead optimization efforts. An alternative scheme that uses SAR matrices for generating virtual compounds was explored in Reference [78]. This work resulted in the development of the COMO [79,80] and DeepCOMO [81] method, which augments the generation of virtual analogues utilizing transfer learning on recurrent neural networks.

Deep learning also has applications in the study of chemical analogues. For example, Peter Ertl showed that a deep neural networks can automatically learn how to propose bioisosteric replacements, mimicking medicinal chemists’ choices [82]. Furthermore, some methods for the exploration of (analogue) chemical space or generation of focused libraries have been proposed on the basis of deep neural networks [72,83,84,85,86] that go beyond the scope of this review.

For the design of analogue compounds, the investigation of substituent fragments and the popularity of analogue sets is of considerable interest. Takeuchi and Bajorath [87,88] investigated the substituent space utilizing CCR analogue series obtained from ChEMBL [10]. For the 500 most popular fragments, preferential replacements were identified and organized in a hierarchical structure.

### 4.2. Structure Activity Relationships and Property Cliffs

In the past, many studies have focused on the the comprehensive analysis of MMPs and their associated transformations in large bioactive databases, such as ChEMBL [10], BindingDB [89], or DrugBank [90]. The efficiency of MMP algorithms for large databases allows the comprehensive identification of (frequent) transformations and scaffold structures without any predefined restrictions on transformation or scaffold type. For example, Wassermann and Bajorath published several papers on the identification of bioisosteric and activity change-inducing transformations obtained from MMPs, respectively [91,92,93]. Hu and Bajorath identified around 300 transformations that were exclusive to MMPs, where molecules had distinct non-overlapping target profiles [94]. In Reference [95], Bemis–Murcko scaffolds were identified for ChEMBL and DrugBank compounds and were investigated for MMP, substructure, and cyclic skeleton relationships. Synthetically feasible RECAP-MMPs only amounted to a small fraction (less than 10%) compared to regular single-cut MMPs, scaffold pairs forming substructure relationships, and those with a common cyclic skeleton. Hu and Bajorath searched for analogues of approved drugs in ChEMBL using different definitions of analogues: MMPs, MCS search, exhaustive fragmentation, and RECAP-MMPs. RECAP-MMPs were scarce when compared against MCS-MMPs (70 versus 671). However, RECAP-MMPs had a higher proportion of overlapping activity profiles. Notably, analyses, such as this one, combining different MMP definitions, produce more comprehensive knowledge from databases. For example, besides the diverse scaffold analysis facilitated by using different MMP definitions, the inclusion of the exhaustive fragmentation algorithm facilitated the study of the most common chemical transformations [94,95].

Other studies have proven the usefulness of RECAP-MMP analogue series in structure-activity relationship (SAR) analysis in several different contexts [96]. Analogue series displaying a consensus in the inhibition patterns were identified with MMPA in high-throughput screening (HTS) assays performed on cancer cell lines [97,98]; this highlights the possibility of identifying cell-selective analogue series by systematic data mining of HTS results.

Activity cliffs (ACs) are defined as “pairs of structurally similar compounds that display a large difference in potency against a given target” [99,100]. This concept can be generalized to “property cliffs”, considering any relevant endpoint not limited to potency [101]. Cliff analysis can provide structural insights that help in rationalizing the activity profile of a set of compounds [102]. Interestingly, ACs are mostly not formed as isolated pairs but occur in clusters, where one or more compounds participate in multiple ACs forming so-called “coordinated” ACs. This points to the identification of clusters of compounds having a higher content of SAR-relevant information [100].

Although different molecular similarity metrics help recognize activity cliffs, the MMP concept lends itself well to an intuitive and easily interpretable definition of so-called MMP-ACs [100]. Some transformations are more likely to produce an activity cliff independent of the biological context. For example, substituting a phenyl ring with an iodine atom is 16 times as likely to form an AC as if fluorine was used instead [93]. Of note, MMP-based approaches do not take three-dimensional features and chirality into account. However, such information might be relevant for AC formation [103], and combining MMP approaches with 3D descriptors has led to the concept of 3D-2D-cliffs, thus extending the concept of purely 3D-cliffs [104].

The third and latest generation of ACs emerged from advances in analogue series identification [105]. The CCR method allows the introduction of new ACs categories: the multi-site, isomeric, and privileged substructure ACs [106]. Multi-site ACs are not as common as single-site ACs. Therefore, single-site ACs might capture most of the relevant SAR knowledge in a chemical library. However, the simultaneous study of multiple substitution sites acknowledges the existence of synergistic modifications, a novelty in AC analysis [107]. The continuous evolution of the AC concept pinpoints the road ahead in SAR analysis through MMP-related approaches [105].

### 4.3. Virtual Screening and ADMET Prediction

Most of the applications mentioned above centered on knowledge extraction from large databases where the endpoints for each compound are known. However, the extraction of MMPs and analogue series does not rely on activity or property annotation, thus making the application of these algorithms attractive for predictive and virtual screening tasks. Similar to lead optimization campaigns, the local SAR of compounds with known activity can be explored through MMP or MMS analysis and can guide the future exploration of promising structures. Moreover, in its most straightforward application, databases can be screened for existing or purchasable analogues based on MMPs or CCRs, thus mimicking the creative exploration of analogue space in synthesis campaigns.

Kanetaka et al. [108] identified a diphenyl ether that inhibits the enoyl-acyl carrier protein reductase (Inh (a) in *Mycobacterium tuberculosis*) by MMP analysis. They identified 32 analogues with single substitutions in a commercial database listing 461,383 compounds (ChemBridge) and used molecular docking to select the top 10 compounds for biological testing and ADMET evaluation. In this way, they presented a thorough exploration of the SAR of this analogue series [108]. Moreover, MMPA-related approaches may also augment virtual screening campaigns. For example, Fu et al. [109] combined QSAR models with MMPA to identify general optimization rules for the distribution coefficient log D [109].

MMP algorithms can also extract ADMET knowledge from databases of commercial interest. By focusing only on the extracted transformations, knowledge transfer in the private sector is possible because chemical structures can remain industrial secrets [110,111].

The automatic extraction of analogue series, specifically MMS, is a source of unique quality for state-of-the-art property prediction algorithms because of the local context of MMP transformations. Compounds can be organized into analogue series allowing the construction of local models for predicting potency, ADMET, and other properties [112]. Strikingly, these models have accuracies comparable to some standard machine-learning procedures [112]. Moreover, standard QSA(P)R methodologies for the study of global SAR can be augmented by including MMP analyses. Thus, the effect of transformations on ADMET properties has been successfully predicted [113]. Combining machine learning, QSA(P)R, and MMP analysis is a promising approach to obtain a balance between generalizability and keeping the applicability domain in sight when modeling ADMET [114].

## 5. Exemplary Sar Analysis with CCR-Based Approaches

**Figure 6:**
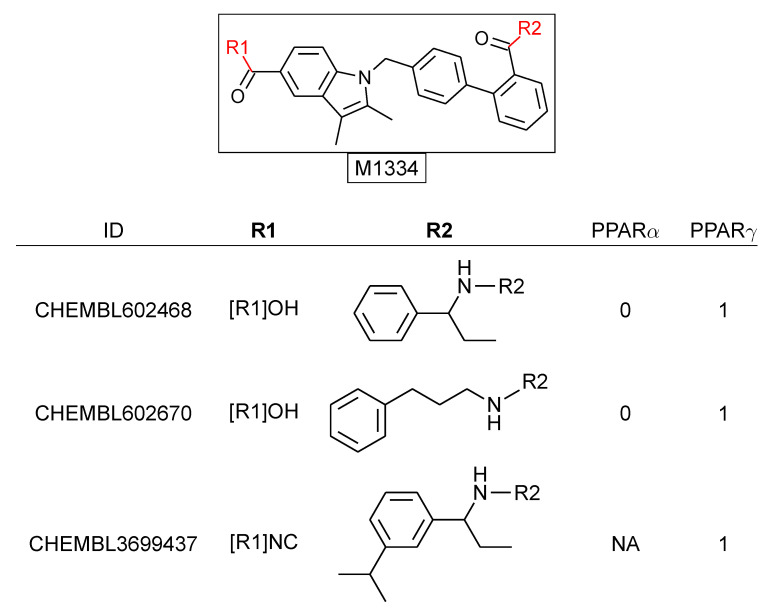
R-group table containing three (of more than a hundred) compounds matching the M1334 core.

**Figure 7:**
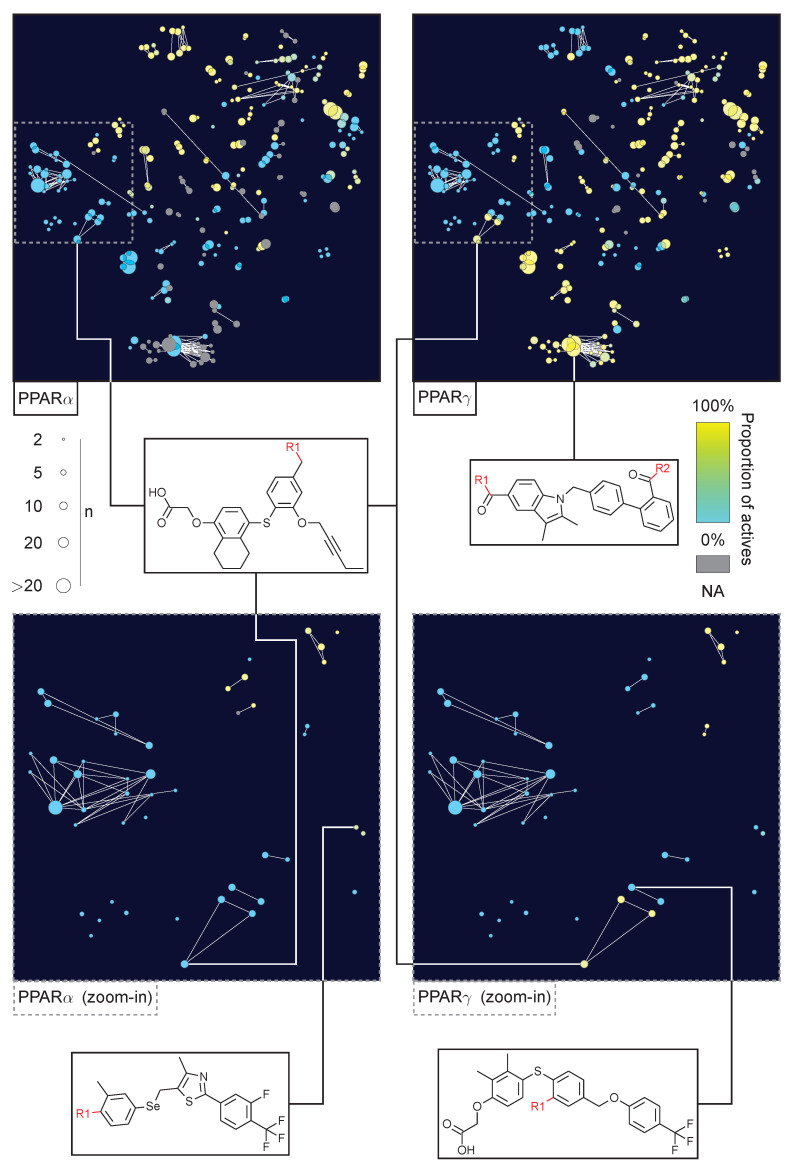
Constellation plot for a library of PPAR agonists. Analogue series are represented as connected dots, where the dots represent core substructures of molecules. The size of a dot corresponds to the number of molecules matching the core structure. Two dots are connected if both cores match at least one common molecule. The core structures are projected onto a two-dimensional plane based on their structural similarity (see Reference [9]).

We consider a set of 3073 molecules with activity reports deposited in ChEMBL 29 [10] against PPARα or PPARγ. Note that 756 and 1323 of the molecules had only information regarding PPARα or PPARγ, respectively, while 994 molecules had annotations for both. Using the RECAP-CCR approach, all molecules were fragmented and indexed. Molecules matching any of the resulting cores can be readily organized into an R-group table (see Figure 6). Figure 7 shows a constellation plot with 153 analogue series with at least three compounds each, comprising 1420 (≈46%) of the molecules in the data set and summarized in 266 cores (colored dots). A limitation of constellation plots is that molecules that didn’t match any analogue series are not considered in the analysis.

**Figure 8:**
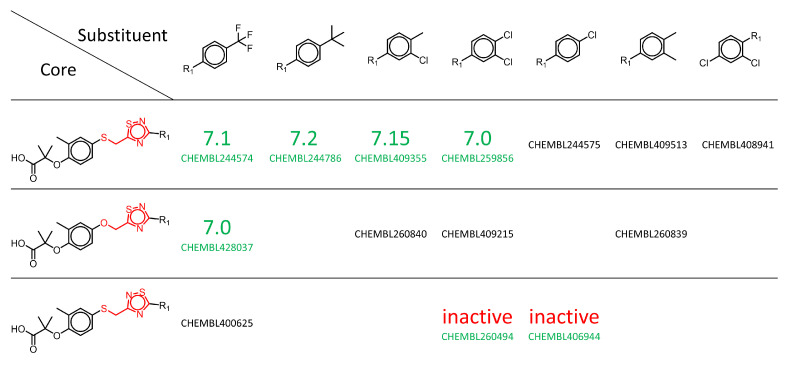
Exemplary SAR matrix. An SAR matrix identified from a data set of PPARα/γ agonists extracted from ChEMBL is shown. Each row represents an MMS. The leftmost column shows the core structures forming a core MMS with the variable part shown in red. The other columns are headed by the substituents. Logarithmic potency values for PPARα are shown in green. Inactive compounds are indicated by the red text. ChEMBL ids in black indicate compounds with no annotation against PPARα. Empty cells represent potential compounds for further SAR exploration that were not part of the data set. With the exception of ChEMBL244574, which did not possess any PPARγ annotation, all other compounds are inactive against PPARγ.

The analysis of the constellation plot in Figure 7 allows the identification of regions in the chemical space, as well as specific analogue series, with dual or selective activity against PPARα and PPARγ. The analysis can be augmented by SAR matrices, which are readily obtained, further enhancing the quality of available SAR insights (Figure 8). The methodologies presented here in an exemplary manner highlight their potential in exploring the SAR of these targets by identifying chemical space regions prone to dual or selective activity, as well as indicating unexplored regions of chemical space that might be promising for further testing.

## 6. Conclusions

The efficient identification of analogue compounds and complete analogue series in compound databases poses a relevant and exciting challenge. Approaches based on rigorous core structure definitions, such as the Bemis–Murcko scaffold, have been among the first to be applied for this purpose. However, analogue series obtained this way would not allow ring-containing substituents since the core structure, by design, includes all the rings of a molecule.

One approach to relax this limitation has been to decompose ring systems, which results in a hierarchical scaffold organization while systematic fragmentation explores every potential core structure per molecule directly in a data-driven approach to group molecules—in pairs, series, or matrices—leading to richer SAR analyses, chemical space exploration, and predictions. The flexibility of the fragment-and-index approach allows retrieved analogue series to focus on synthetic accessibility or on more general structural similarity. The former is particularly valuable when virtual analogue compounds are considered for further exploration, while the latter is better suited for retrospective SAR analysis in detecting activity patterns or property-influencing rules. Nonetheless, the fragment-and-index approach still poses restrictions on the type of analogue series that can be detected, as no variation in the core structure is allowed. Therefore, it is unlikely to detect MMPs with minor modifications, such as sulfur or oxygen substitutions, in the core structure. Furthermore, core structures might be represented with different tautomeric structures, and indexing will not necessarily recognize them as identical. Nevertheless, the automatic identification of analogue series has reached considerable methodological maturity, enabling the efficient and comprehensive processing of large compound databases.

We highlighted several applications of automatic analogue series identification to aid SAR analysis and drug design, and we expect the synergistic effect that the identification of analogue compound series from diverse sources can have for SAR elucidation and exploration will become more and more relevant with the increasing size of annotated compound databases. A key advantage of the methodologies explored here is the chemical interpretability of extracted SARs. This is in contrast to deep learning approaches in cheminformatics that are characterized by their “black box” nature. However, these methodologies are not mutually exclusive, as some recent publications have shown [73,81,82]. The comprehensive analysis of the analogue space of compound databases paired with deep learning models constitutes a fruitful basis for further methodological developments for prediction and classification tasks, as well as for the exploration of analogue chemical space.

## Figures and Tables

**Table 1:** Comparison of common MMP definitions.

MMP Definition	Concept	Advantages	Disadvantages	References
Transformation-based	Only bonds matching a transformation can be cut.	Computationally efficient. Chemically meaningful transformations are studied.	Limited to a set of predefined transformations. Only pairwise comparisons.	[21,24,25,30]
MCS-based	Topological identification of the maximum common substructure between molecules.	Exhaustive. Can extract specific transformations.	High computational complexity.	[18,19]
Fragmentation-based (exhaustive)	Every acyclic single bond can be cut. Two molecules form an MMP if they can be reduced to a common substructure of significant size.	Computationally efficient for large databases using the fragment and index approach. No predefined transformations limit the algorithm. Compounds can be organized in analogue series. Yields scaffolds and transformations.	Chemical feasibility of the generated cuts and transformation is not considered. Inefficient for pairwise comparisons. Algorithmic limitations on core structures are imposed. Systematic fragmentation can be time consuming for some large molecules.	[14]
Fragmentation-based using retrosynthetic rules	Bonds are cut according to retrosynthetic rules. Two molecules form an MMP if they can be reduced to a common substructure of significant size.	Computationally efficient for large databases using the fragment and index approach. Chemically meaningful core structures shared by MMPs. Compounds can be organized in analogue series. Hierarchical organization of analogue series is possible.	Limited to the list of retrosynthetic rules. Inefficient for pairwise comparisons. Algorithmic limitations on core structures are imposed.	[2,3,36,37]

## Abbreviations

The following abbreviations are used in this manuscript:

ADMET	absorption, distribution, metabolism, elimination, toxicity
ASBS	analogue series-based scaffold
BMMSG	bipartite matched molecular series graph
CCR	compound-core relationship
CReM	chemical reasonable mutations
CSN	chemical space networks
MMP	matched molecular pair
MMPA	matched molecular pair analysis
MMS	matched/matching molecular series
QSA(P)R	quantitative structure activity (property) relationships
RECAP	retrosynthetic combinatorial analysis procedure
SAR	structure-activity relationships
SRP	single R-group polymorphisms
